# Relationship of compartment-specific structural knee status at baseline with change in cartilage morphology: a prospective observational study using data from the osteoarthritis initiative

**DOI:** 10.1186/ar2732

**Published:** 2009-06-17

**Authors:** Felix Eckstein, Wolfgang Wirth, Martin I Hudelmaier, Susanne Maschek, Wolfgang Hitzl, Bradley T Wyman, Michael Nevitt, Marie-Pierre Hellio Le Graverand, David Hunter, the OA Initiative Investigator Group

**Affiliations:** 1 Institute of Anatomy and Musculoskeletal Research, Paracelsus Medical University, Strubergasse 21, A5020 Salzburg, Austria; 2 Chondrometrics GmbH, Ulrichshöglerstrasse 23, D83404 Ainring, Germany; 3 Research Office, Paracelsus Medical University, Strubergasse 21, A 5020 Salzburg, Austria; 4 Pfizer Global Research and Development, 50 Pequot Ave, New London, CT 06320, USA; 5 University of California and OA Initiative Coordinating Center, 185 Berry Street, San Francisco, CA 94107, USA; 6 Division of Research, New England Baptist Hospital, 125 Parker Hill Avenue, Boston, MA 02120, USA

## Abstract

**Introduction:**

The aim was to investigate the relationship of cartilage loss (change in medial femorotibial cartilage thickness measured with magnetic resonance imaging (MRI)) with compartment-specific baseline radiographic findings and MRI cartilage morphometry features, and to identify which baseline features can be used for stratification of fast progressors.

**Methods:**

An age and gender stratified subsample of the osteoarthritis (OA) initiative progression subcohort (79 women; 77 men; age 60.9 ± 9.9 years; body mass index (BMI) 30.3 ± 4.7) with symptomatic, radiographic OA in at least one knee was studied. Baseline fixed flexion radiographs were read centrally and adjudicated, and cartilage morphometry was performed at baseline and at one year follow-up from coronal FLASH 3 Tesla MR images of the right knee.

**Results:**

Osteophyte status at baseline was not associated with medial cartilage loss. Knees with medial joint space narrowing tended to show higher rates of change than those without, but the relationship was not statistically significant. Knees with medial femoral subchondral bone sclerosis (radiography), medial denuded subchondral bone areas (MRI), and low cartilage thickness (MRI) at baseline displayed significantly higher cartilage loss than those without, both with and without adjusting for age, sex, and BMI. Participants with denuded subchondral bone showed a standardized response mean of up to -0.64 versus -0.33 for the entire subcohort.

**Conclusions:**

The results indicate that radiographic and MRI cartilage morphometry features suggestive of advanced disease appear to be associated with greater cartilage loss. These features may be suited for selecting patients with a higher likelihood of fast progression in studies that attempt to demonstrate the cartilage-preserving effect of disease-modifying osteoarthritis drugs.

## Introduction

The Osteoarthritis (OA) Initiative is a program targeted at characterizing risk factors associated with the onset and progression of symptomatic knee OA and at identifying sensitive biomarkers of symptomatic knee OA. To this end, fixed flexion radiography [1-5] and 3 Tesla magnetic resonance imaging (MRI) scans [6-8] were performed at baseline and at regular follow-up in 4796 participants. We [9,10] and others [11] have recently reported a modest (but significant) change in femorotibial cartilage morphology over one year in the first publicly released longitudinal data in an age- and gender-stratified subcohort of 160 participants [12]. Potential predisposing factors of subsequent cartilage loss, including age, sex, body mass index (BMI), symptom status, and the Kellgren Lawrence grade (KLG), were assessed. Cartilage loss was found to be more prominent in the medial (than in the lateral) femorotibial compartment, and more prominent in the weight-bearing femur than in the tibia [9-11]. Participants with high BMI and radiographic OA (as determined by the KLG [13]) were observed to display trends toward higher rates of change than those with lower BMI and without radiographic OA [9], although the relation failed to reach statistical significance. The limitation of the KLG [13], however, is that it mixes distinct constructs (osteophytes, joint space narrowing (JSN), subchondral sclerosis, subchondral bone shape changes, cysts, etc) into one scale with the invalid assumptions that changes are linear [14].

In addition, the KLG is not specific to the medial or lateral femorotibial compartment. The association of compartment-specific baseline MRI cartilage morphology measures with longitudinal cartilage loss has not been previously investigated in this cohort. An accurate stratification during study recruitment with regard to 'progressors' with relatively rapid cartilage loss is, however, important, because the potential therapeutic effect of a disease-modifying OA drug can be demonstrated using much lower sample sizes if only participants with a high likelihood of fast progression are recruited.

The objective of the current study was therefore to determine whether compartment-specific individual radiographic features (JSN, osteophytes, sclerosis, and others) at baseline, and compartment-specific structural status of the knee cartilage in MRI (specifically denuded subchondral bone area and cartilage thickness) are predictive of longitudinal medial femorotibial cartilage thickness loss over one year.

## Materials and methods

An age- and gender-stratified subsample (OA Initiative public-use datasets 0.1.1, 0.B.1, and 1.B.1) of the OA Initiative progression subcohort was studied, with the exclusion criteria (i.e. rheumatoid or inflammatory arthritis, bilateral end stage knee OA, inability to walk without aids, and 3 Tesla MRI contraindications) and other details having been described previously [9]. The OA Initiative is conducted in compliance with the ethical principles derived from the Declaration of Helsinki and in compliance with local Institutional Review Board, informed consent regulations, and International Conference on Harmonization Good Clinical Practices Guidelines.

The subsample studied included 79 women with a mean ± standard deviation age of 60.3 ± 9.5 years and BMI of 30.3 ± 5.5, and 77 men with an age of 62.0 ± 10.2 years and BMI of 30.1 ± 3.7. The age range examined was 45 to 79 years. All participants had frequent knee symptoms, and radiographic OA, as defined by definite osteophytes in the postero-anterior fixed flexion radiographs [1,2] in at least one knee from the clinical site readings.

The current analysis relied on the results of the independent radiographic readings by a musculoskeletal radiologist and rheumatologist, which in cases of discrepancy were adjudicated by consensus with a third reader. The following features were graded on the baseline radiograph based on the Osteoarthritis Research Society International atlas: medial and lateral JSN (graded 0 to 3) [15,16]; medial and lateral tibial and femoral osteophytes (graded 0 to 3) [15,16]; medial and lateral tibial and femoral subchondral sclerosis (graded 0 to 3) [15,16]; medial and lateral tibial subchondral bone attrition (graded 0 to 3) [15,16]; medial and lateral tibial and femoral cysts (graded 0 or 1); medial and lateral compartment chondrocalcinosis (graded 0 or 1).

The MRI sequence used to quantify cartilage morphology (see below) was only acquired in the right knees [17], whereas some participants displayed symptoms and radiographic OA in their left knee. As a result of this and because the adjudicated central radiographic readings may have differed from the initial screening readings, not all knees analyzed had radiographic (or symptomatic) knee OA. Of the 156 right knees analyzed, 17 (11%) were KLG 0, 29 (19%) KLG1, 56 (36%) KLG 2, 47 (30%) KLG 3, and 7 (4%) were KLG 4.

**Figure 1:**
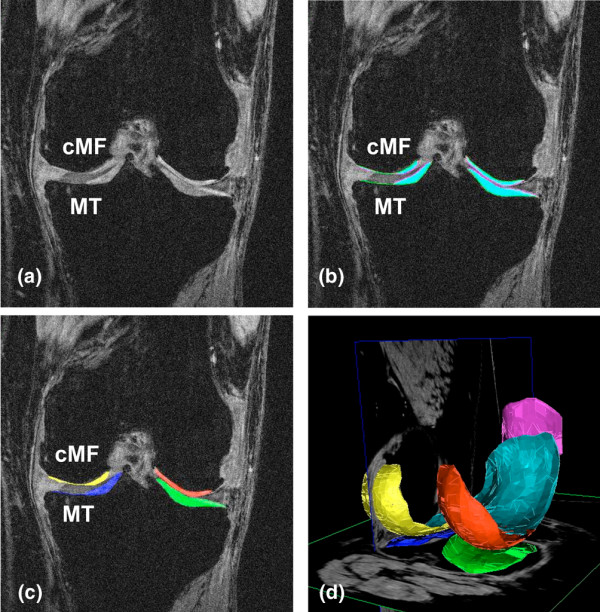
Knee magnetic resonance image obtained with fast low angle shot (FLASH) sequences with water excitation. **(a) **Double oblique coronal image showing the regions of interest used in the current analysis. **(b) **Same coronal image with the total area of subchondral bone (tAB) being segmented in green, the area of the cartilage surface (AC) in magenta, and the filling between the two surfaces in turquoise. Note the denuded area (tAB not covered by AC) in the medial tibia and the medial femur. **(c) **Same coronal image with the medial tibial (MT) cartilage marked (segmented) blue, weight-bearing medial femoral cartilage (cMF) marked yellow, the lateral tibial cartilage marked green, and the lateral weight-bearing femoral cartilage marked red. **(d) **3D reconstructions of knee cartilage plates from a sagittal data set in a different person: The femoro-tibial cartilages are labeled with the same colors as in (c), the patellar cartilage is labeled magenta and the trochlear (femoral) cartilage in turquoise.

MRI was performed using four 3 Tesla scanners (Siemens Magnetom Trio, Erlangen, Germany) and quadrature transmit-receive knee coils (USA Instruments, Aurora, OH, USA). Double oblique coronal 3D fast low angle shot (FLASH) MRI with water excitation were acquired as described previously [6-9] (Figure 1). After initial quality control at the image analysis center (Chondrometrics GmbH, Ainring, Germany), manual segmentation of the femorotibial cartilages was performed by seven technicians with at least three years of experience in cartilage segmentation [6-9,18] (Figure 1). The image data were processed in pairs (baseline and one year follow up), the readers being blinded to the order of the image acquisition. Because of the higher rate of change in the medial compartment that was reported previously [9,11], and because of the relatively few subjects with lateral radiographic femorotibial OA (see below), medial cartilage loss was used as an outcome measure (Figure 1). However, knees with predominantly lateral radiographic OA were not eliminated from the study. Specifically, we analyzed one year changes in the mean cartilage thickness over the total subchondral bone area (ThCtAB) in the medial tibia (MT), central (weight bearing) medial femoral condyle (cMF [6,7]; Figure 1), and medial femorotibial compartment (MFTC = summed values of MT and cMF).

**Figure 2:**
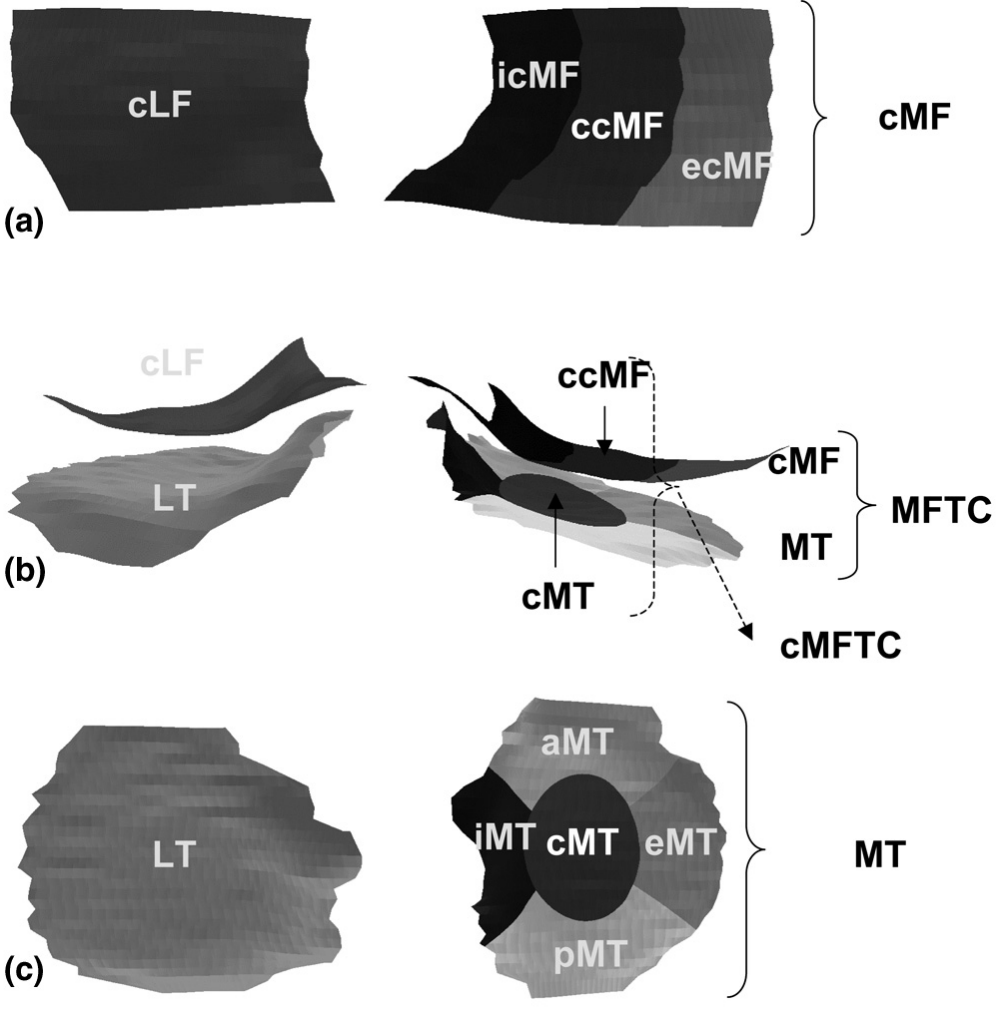
Cartilage plates and subregions used as outcome measures in this study. **(a) **Inferior view of the weight-bearing (central) part of the medial (cMF) and lateral femoral condyle (cLF). ccMF = central third of cMF (icMF and ecMF = internal and external third of cMF, respectively). **(b) **Posterior view of the femorotibial joint. MFTC = cMF + MT; cMFTC = ccMF + cMT. **(c) **Superior view of the medial (MT) and lateraltibia (LT). cMT = central part of MT (iMT, eMT, aMT, and pMT = internal, external, anterior, and posterior parts of MT, respectively).

As changes in central subregions of the above plates were shown to display greater changes than the total cartilage plates in this subcohort [10], we also considered the central tibia (cMT, covering the central 20% of the subchondral bone area), the central weight-bearing medial femur (ccMF, covering 33%) and the central MFTC (cMFTC = sum of cMT and ccMF) as outcome measures [19] (Figure 2). The mean change, standard deviation of change, standardized response mean (SRM), and the significance of the change (two sided paired t-test, without correction for multiple testing) were calculated for each measure. The SRM provides a measure of the 'sensitivity to change' and was computed by dividing mean change in a given group or subgroup by the standard deviation of the change in this group or subgroup. A negative SRM expresses cartilage loss, and the greater (more negative) the SRM, the greater and more uniform is the cartilage loss in a given group or subgroup. The mean percentage change was calculated by relating the mean change (in mm) in all knees to the mean baseline values of all knees.

The following baseline features of quantitative MRI of cartilage morphology were examined in the context of predicting change: mean cartilage thickness over the total subchondral bone area (ThCtAB) in MT, cMF, and MFTC, and presence of a denuded area of more than 1% of the total subchondral bone area in MT, cMF, or MFTC. Percentage values in MT and cMF were added to provide a measure of denuded bone area in MFTC. The cut-off value of more than 1% was chosen to exclude small areas of an uncovered total area of subchondral bone (three cases in MT, no case in cMF) that may potentially result from small imaging artifacts.

Analysis of variance (ANOVA) was used first to test whether categorical features of structural knee status in fixed flexion radiographs or MRI cartilage morphometry (JSN, osteophytes, subchondral sclerosis, presence of denuded areas of subchondral bone) were significantly associated with cartilage loss over one year, as measured in absolute thickness change (mm). General linear models (GLM) were then used to assess the relation of these features with cartilage loss after adjusting for age, sex, and BMI, and to test whether the continuous variable 'ThCtAB at baseline' was significantly associated with cartilage loss.

## Results

### Structural changes in fixed flexion radiography at baseline

The frequency of radiographic features of OA present in this subcohort is displayed in Table 1. Only a few participants showed bone cysts, chondrocalcinosis, or subchondral bone attrition, and these features were therefore not considered for further analysis.

**Table 1:** Frequency (n) of structural abnormalities on adjudicated reading of baseline fixed flexion radiographs

	Grade 0	Grade 1	Grade 2	Grade 3
Medial joint space narrowing	70	46	35	5
Lateral joint space narrowing	130	14	10	2
Medial tibia osteophyte	51	73	23	9
Medial femoral osteophyte	77	20	20	39
Medial subchondral tibial sclerosis	103	32	17	4
Medial subchondral femoral sclerosis	100	35	16	5
Medial tibial cysts	142	14	-	-
Medial femoral cysts	155	1	-	-
Medial chondrocalcinosis	139	17	-	-
Medial tibial bone attrition	154	2	-	-

Of 70 knees without medial JSN (mJSN), 32 (46%) had no medial (tibial or femoral) osteophytes and 38 (64%) displayed medial osteophytes. Of 86 knees with mJSN, 76 (88%) also displayed definite osteophytes and 10 (12%) did not. Lateral JSN was less frequent than medial JSN (Table 1). In 80 knees (51%), the mJSN grade was higher than the lateral JSN grade, in 56 (36%) it was the same, and in 20 (13%) the lateral JSN grade was higher than the medial compartment.

When taking the maximal osteophyte score of the medial tibia and femur in each subject, 42 subjects (27%) had no medial tibiofemoral osteophyte, 53 (34%) displayed grade 1 osteophytes, 21 (13%) grade 2 osteophytes, and 40 (26%) grade 3 osteophytes.

### Relation of structural changes in fixed flexion radiography at baseline with longitudinal changes in cartilage thickness (cartilage loss)

The rate of change, sensitivity to change (SRM), and significance of the change in cartilage thickness (ThCtAB) for the MFTC is shown in Table 2. Across all 156 subjects, the greatest (most negative) SRM (-0.33) was observed in the cMFTC. The mean change and SRM of cMFTC tended to be greater in knees with the presence of medial JSN (grades 1 to 3: -2.4%; -0.44) than in those without JSN (-1.1%; -0.22), but the difference did not attain statistical significance in the univariate (ANOVA) or multifactorial (GLM) analysis (*P *= 0.66 and r^2 ^= 2.0% after adjusting for age, sex, and BMI). Age, sex, and BMI were no significant predictors in the multifactorial model. This also applied to the other medial cartilage plates and subregions (Table 2). The relatively greatest change (-5.6%; ccMF) and SRM (-0.50; cMF) was found in participants with grade 2 or 3 medial JSN (Table 2). No significant effect of the presence of lateral JSN at baseline was observed on the rate of change in the medial compartment.

**Table 2:** Change in cartilage thickness (ThCtAB) over one year in all participants and in participants with and without medial femoro-tibial joint space narrowing (mJSN) at baseline

	All (n = 156)			mJSN 0 (n = 70)			mJSN 1 (n = 46)			mJSN 2 or 3 (n = 40)		
				
	MC%	SRM	*P *(FUvs.BL)	MC%	SRM	*P *(FUvs.BL)	MC%	SRM	*P *(FUvs.BL)	MC%	SRM	*P *(FUvs.BL)
MT	-0.5	-0.16	0.04274	-0.2	-0.09	0.4554	-0.7	-0.23	0.1215	-0.9	-0.20	0.2115
cMF	-1.9	-0.30	0.00021	-1.0	-0.19	0.1244	-1.8	-0.31	0.0399	-4.4	-0.50	0.0033
MFTC	-1.2	-0.31	0.00015	-0.7	-0.19	0.1231	-1.3	-0.38	0.0134	-2.5	-0.45	0.0069
cMT	-0.9	-0.20	0.01505	-0.3	-0.08	0.4819	-1.0	-0.25	0.0975	-1.8	-0.31	0.0606
ccMF	-2.8	-0.31	0.00013	-1.9	-0.23	0.0537	-2.8	-0.34	0.0274	-5.6	-0.45	0.0066
cMFTC	-1.7	-0.33	0.00005	-1.1	-0.22	0.0704	-1.9	-0.40	0.0090	-3.2	-0.47	0.0046

The *P *value indicates the level of significance for changes between year one follow up (FU) versus baseline (BL) data using a paired t-test.

ccMF = central subregion of the weight bearing medial femoral condyle; cMF = weight bearing medial femoral condyle; cMFTC = central medial femorotibial compartment; cMT = central subregion of the medial tibia; MC% = mean change (in %); MFTC = medial femorotibial compartment; MT = medial tibia; SRM = standardized response mean (mean change/SD of change).

Participants with and without medial femorotibial osteophytes displayed no significant differences in the rate and sensitivity to change in cartilage thickness (Table 3). The same observation applied to the presence and absence of lateral osteophytes (data not shown).

**Table 3:** Change in cartilage thickness (ThCtAB) over one year in participants with various grades of medial femorotibial osteophytes (mOP) at baseline

	mOP 0 (n = 42)			mOP 1 (n = 53)			mOP 2 (n = 21)			mOP 3 (n = 40)		
				
	MC%	SRM	*P *(FUvs.BL)	MC%	SRM	*P *(FUvs.BL)	MC%	SRM	*P *(FUvs.BL)	MC%	SRM	*P *(FUvs.BL)
MT	-1.1	-0.46	0.00475	0.0	0.01	0.94330	-1.0	-0.24	0.28311	-0.3	-0.10	0.52858
cMF	-1.9	-0.35	0.02844	-1. 7	-0.31	0.02878	-2.6	-0.40	0.08254	-1.8	-0.21	0.18398
MFTC	-1.5	-0.46	0.00509	-0.9	-0.23	0.10443	-1.8	-0.39	0.09264	-1.0	-0.24	0.14224
cMT	-1.5	-0.42	0.00889	0.0	-0.01	0.94955	-1.7	-0.31	0.16985	-0.7	-0.15	0.35837
ccMF	-2.6	-0.33	0.04059	-2.9	-0.40	0.00582	-3.5	-0.36	0.11512	-2.3	-0.20	0.21479
cMFTC	-2.1	-0.42	0.00987	-1.4	-0.31	0.02639	-2.5	-0.39	0.08638	-1.4	-0.24	0.14412

The *P *value indicates the level of significance for changes between year one follow up (FU) versus baseline (BL) data using a paired t-test.

ccMF = central subregion of the weight bearing medial femoral condyle; cMF = weight bearing medial femoral condyle; cMFTC = central medial femorotibial compartment; cMT = central subregion of the medial tibia; MC% = mean change (in %); MFTC = medial femorotibial compartment; MT = medial tibia; SRM = standardized response mean (mean change/SD of change).

Knees with medial femoral subchondral bone sclerosis showed significantly greater cartilage loss in the univariate and multifactorial analyses (up to -6.6% and SRMs up to -0.48; Table 4) in cMF (*P *< 0.05; r^2 ^= 5.7%), ccMF (*P *< 0.05; r^2 ^= 4.7%), MFTC (*P *< 0.05; r^2 ^= 5.3%), and cMFTC (*P *< 0.05; r^2 ^= 4.5%) than those without sclerosis (up to -1.4% and SRM = -0.23; Table 4). Again, age, sex, and BMI did not make a significant contribution in the GLM. Knees with medial tibial subchondral bone sclerosis also generally displayed greater cartilage loss than those without, but the difference did not reach statistical significance (Table 4). No significant differences in progression were detected between knees with and without lateral tibial or femoral subchondral bone sclerosis (data not shown).

**Table 4:** Change in cartilage thickness (ThCtAB) over one year in participants with and without medial subchondral bone sclerosis at baseline

	No tibial sclerosis (n = 103)			Tibial sclerosis (n = 53)			No femoral sclerosis (n = 100)			Femoral sclerosis (n = 56)		
				
	MC%	SRM	*P *(FUvs.BL)	MC%	SRM	*P *(FUvs.BL)	MC%	SRM	*P *(FUvs.BL)	MC%	SRM	*P *(FUvs.BL)
MT	-0.3	-0.13	0.19393	-0.9	-0.22	0.12093	-0.3	-0.11	0.26937	-1.0	-0.24	0.08337
cMF	-1.4	-0.23	0.02012	-3.2	-0.46	0.00168	-0.8	-0. 17	0.08584	-4.6	-0.48	0.00064
MFTC	-0.9	-0.24	0.01704	-2. 0	-0.46	0.00168	-0.6	-0.19	0.05783	-2.7	-0.48	0.00074
cMT	-0.5	-0.14	0.15907	-1.6	-0.29	0.04127	-0.4	-0.12	0.22676	-1.7	-0.31	0.02597
ccMF	-2.3	-0.27	0.00678	-4.2	-0.42	0.00369	-1.4	-0.23	0.02596	-6.6	-0.44	0.00171
cMFTC	-1.4	-0.27	0.00639	-2.7	-0.46	0.00141	-0.9	-0.24	0.01730	-3.7	-0.47	0.00094

The *P *value indicates the level of significance for changes between year one follow up (FU) versus baseline (BL) data using a paired t-test.

ccMF = central subregion of the weight bearing medial femoral condyle; cMF = weight bearing medial femoral condyle; cMFTC = central medial femorotibial compartment; cMT = central subregion of the medial tibia; MC% = mean change (in %); MFTC = medial femorotibial compartment; MT = medial tibia; SRM = standardized response mean (mean change/SD of change).

### Structural changes in MRI at baseline

Thirty-four participants (22%) displayed a tibial denuded area in MRI (>1%), with the size ranging from 1.3% to 32% of the total area of subchondral bone (median = 10.7%). Thirty-six participants (23%) showed a femoral denuded area that ranged from 1.6% to 65% (median = 10.9%). Fifty knees (32%) displayed either tibial or femoral, and 20 knees (13%) both tibial and femoral denuded area.

### Relation of structural changes in MRI at baseline with longitudinal changes in cartilage thickness (cartilage loss)

Knees with a denuded area in MFTC showed a significantly greater cartilage loss in MT (*P *< 0.01; r^2 ^= 6.4%) and cMT (*P *< 0.001; r^2 ^= 8.0%) than knees without denuded areas (Table 5). Knees with denuded areas in cMF also displayed significantly greater cartilage loss in MT (*P *< 0.05; r^2 ^= 5.3%) and cMT (*P *< 0.001; r^2 ^= 8.4%) than those without a denuded area in cMF. These observations were consistent in the univariate and multifactorial analyses. No significant differences were observed for knees with and without denuded areas in MT, or for other outcomes. The greatest SRM (-0.64) was observed for the change in cMFTC in subjects with a denuded area in cMF (Table 5).

**Table 5:** Change in cartilage thickness (ThCtAB) over one year in participants with without and with more than 1% denuded area (dAB) in the medial tibia or the medial weight-bearing femoral condyle at baseline

	No MFTC.dAB > 1% (n = 106)			MFTC.dAB > 1% (n = 50)			MT.dAB > 1% (n = 37)			cMF.dAB > 1% (n = 36)		
				
	MC%	SRM	*P *(FUvs.BL)	MC%	SRM	*P *(FUvs.BL)	MC%	SRM	*P *(FUvs.BL)	MC%	SRM	*P *(FUvs.BL)
MT	-0.1	-0.04	0.69131	-1.6	-0.43	0.00602	-1.6	-0.42	0.02582	-1.9	-0.51	0.00539
cMF	-1.9	-0.34	0.00073	-2.2	-0.25	0.09440	-1.4	-0.15	0.41551	-4.4	-0.51	0.00540
MFTC	-1.0	-0.30	0.00256	-1.9	-0.35	0.02198	-1.5	-0.27	0.14541	-3.0	-0.57	0.00232
cMT	-0.3	-0.07	0.47903	-2.4	-0.46	0.00318	-2.5	-0.42	0.02605	-3.4	-0.62	0.00105
ccMF	-2.9	-0.35	0.00044	-2.7	-0.23	0.12063	-1.8	-0.14	0.44502	-5.4	-0.47	0. 01001
cMFTC	-1.5	-0.32	0.00134	-2.5	-0.38	0.01261	-2.2	-0.29	0.11271	-4.2	-0.64	0.00074

The *P *value indicates the level of significance for changes between year one follow up (FU) versus baseline (BL) data using a paired t-test.

ccMF = central subregion of the weight bearing medial femoral condyle; cMF = weight bearing medial femoral condyle; cMFTC = central medial femorotibial compartment; cMT = central subregion of the medial tibia; MC% = mean change (in %); MFTC = medial femorotibial compartment; MT = medial tibia; SRM = standardized response mean (mean change/SD of change).

Smaller baseline cartilage thickness in MT was significantly associated with greater cartilage loss in MT (*P *< 0.05; r^2 ^= 2.9%) and cMT (*P *< 0.05; r^2 ^= 3.8%), and smaller baseline cartilage thickness in cMF was significantly associated with greater cartilage loss in cMF (*P *< 0.05; r^2 ^= 3.5%). Thin cartilage in both MT and cMF (MFTC) was significantly associated with greater cartilage loss in cMT (*P *< 0.05; r^2 ^= 4.2%) after adjusting for age, sex, and BMI in the GLM.

## Discussion

This study investigates the relation of compartment-specific structural radiographic knee and MRI cartilage status at baseline with medial femorotibial cartilage thickness loss over one year as measured by 3 Tesla MRI. The results indicate that knees with more advanced medial femorotibial disease display greater cartilage loss than those with less advanced disease. Osteophytes, which represent early radiographic features of OA, did not predict cartilage loss in the current study, but knees with medial JSN tended to exhibit greater cartilage loss in the MFTC than those without JSN, although the relation was not significant. Subchondral femoral sclerosis (radiography), denuded subchondral bone area (MRI), and lower cartilage thickness (in MRI) at baseline displayed significant relations with at least some of the outcome measures of medial cartilage loss, all of these representing measures of relatively advanced disease. Whereas in the entire cohort the greatest SRM was -0.33 (change in cMFTC), the SRM was -0.47 in knees with grades 2/3 medial JSN, -0.46/-0.47 in knees with tibial and femoral subchondral sclerosis, and -0.64 in knees with denuded areas in the weight-bearing femur.

The subcohort examined here was the first one made public by the OA Initiative. It represents a stratified random sample of subjects with complete baseline and 12-month imaging data available as of April 2006, with strata roughly equal by gender and clinical/imaging site. Although it was not intended to be a random sample of the entire progression subcohort, the156 subjects analyzed show baseline characteristics similar to the entire OA Initiative progression subcohort (n = 1389; age range 45 to 79 years; 57% women with an age of 61.5 ± 8.9 years and a BMI of 30.8 ± 5.4; 43% men with an age of 61.1 ± 9.3 years and a BMI of 29.8 ± 4.1). A breakdown of calculated KLGs for all right knees for the entire progression subcohort in comparison with the subsample investigated here (2% vs. 11% KLG 0, 13% vs. 19% KLG1, 31% vs. 36% KLG 2, 39% vs. 30% KLG 3, and 15% vs. 4% KLG 4) shows that the current sample included more cases with no or possible radiographic OA (KLG0/1) and fewer cases with severe radiographic OA (KLG4) than the right knees of the entire progression subcohort, but both samples span all grades of radiographic OA.

Although a larger set of image data has been made public by the OA Initiative, central and compartment-specific radiographic readings (on which these analyses relied) have so far only been performed for the subsample used in this analysis. The statistical power of the current study is therefore limited by the relatively small sample size, which also introduces the potential problem of type 2 error, given the relatively large number of features examined. However, the features examined have been analyzed, but were not interpreted in isolation, in that most of the features found to be associated with higher rates of cartilage loss and greater SRMs were features of advanced structural disease. The current results should nevertheless be viewed as exploratory and must be confirmed in another (larger) sample, before recruitment approaches are based on any of the radiographic or MRI features investigated here. Also, the specific radiographic and MRI features studied must be seen in the context of other potentially predisposing risk factors, such as meniscal extrusion and damage [20-22], bone marrow alterations [21-23], focal cartilage defects [24,25], and limb alignment [26-30], as some of these may be directly or indirectly associated with the described compartment-specific radiographic or MRI features.

Additionally, there exist other potential predisposing factors of structural progression, for which less clear evidence or even contradictory results have been provided, including pain, joint function, physical activity levels [31], synovitis (effusion) [32], sex hormone levels [33,34], and serum or urine biomarkers [35], which eventually need to be taken into account. Another limitation of the study is that, given the limited number of cases and the greater progression observed in the MFTC in this subcohort [9-11], the analysis was deliberately limited to MFTC outcomes, whereas some participants also showed progression in the lateral compartment. Only femorotibial, but not femoropatellar, radiographic features were analyzed, again because of the limited sample size, and because no femoropatellar readings have yet been provided by the OA Initiative. Only the right knee was analyzed, because the MRI sequence used for cartilage morphometry (FLASH) was only acquired in the right, but not in the left knees [17], so that not all knees displayed radiographic and/or symptomatic OA. This, however, allowed us to examine the potential predictive value of radiographic and MRI features across knees with a relatively wide range of radiographic status. The decision to analyze the FLASH sequence acquired in all right knees, rather than the DESS sequence that was available for both knees, was made to allow to directly link the results to previous findings in other cohorts, which have to date been based on FLASH or SPGR sequences.

The strength of the current study is that it uses validated quantitative MRI technology as a measure of structural disease progression, which has been shown to be more powerful in revealing risk factor associations than semi-quantitative scoring of cartilage status [28]. Cartilage loss, as measured with MRI over relatively short periods (1 or 2 years), has been shown to be associated with cartilage loss over longer periods (4.5 years) [36] and with a clinical outcome of OA, specifically knee arthroplasty [37], which makes it a very promising surrogate endpoint. Also, the current analysis relied on central radiographic readings of the OA Initiative imaging data, which was adjudicated in case of discrepancy between two independent readers, and has been shown to deviate in a substantial number of cases from the site readings used for the purpose of recruiting participants for the OA Initiative [11]. Despite the many structural features examined and lack of statistical significance for some of these, the features suggestive of advanced disease pointed towards greater longitudinal cartilage loss compared with knees with less advanced disease.

Felson and colleagues [38] reported that higher osteophyte scores modestly increased the risk of OA progression (defined by increasing JSN over 30 months), in particular when compartment-specific relations were analyzed. The authors mentioned that this association became weaker to non-significant when adjusting for limb alignment. Wolfe and Lane [39] reported that JSN at baseline was a strong predictor of OA progression (defined by advancing to radiographic JSN grade 3) in more than 1500 patients, whereas BMI and osteophytes were less predictive and only contributed in participants with no JSN at baseline. Mazzuca and colleagues [40] also found the progression of JSN over 30 months to be inversely related to baseline joint space width (JSW) and Le Graverand and colleagues [41] the reduction in JSW to be greater in knees with JSN at baseline, using fluoroscopically standardized knee radiographs. In contrast to these and our current findings, Bruyere and colleagues [42] reported patients in the highest quartile of baseline JSW to experience more severe cartilage loss than those in the lowest quartile and thus recommended the inclusion of participants with less severe OA (high JSW, no JSN) in disease-modifying OA drug trials.

Few studies have so far investigated the relationship between MRI-based cartilage loss and radiographic features of OA at baseline. Raynauld and colleagues [21] found no significant association of MRI-based cartilage loss over 24 months with the radiographic JSW at baseline. However, another analysis from the same group [22] reported that cartilage loss in the central aspects of the femorotibial cartilage plates was associated with smaller JSW and higher grades of JSN at baseline, and the results of our current analysis are in agreement with these findings. Wluka and colleagues [43] reported the initial cartilage volume (in MRI) to be the most significant determinant of tibial cartilage loss (volume change with MRI), participants with high initial cartilage volume experiencing more severe cartilage loss than those with smaller cartilage volumes at baseline. Our current results are in direct disagreement with these observations in that we find a greater cartilage loss in participants with low initial cartilage thickness and with already denuded subchondral bone at baseline.

To our knowledge, no previous study has identified a relation between subchondral bone sclerosis at baseline and MRI-based cartilage loss. Buckland-Wright [44] observed that the subchondral cortical plate and adjacent trabeculae thicken in OA, often prior to the onset of JSN. However, it was also reported that sclerosis did not increase in knees until the medial JSW was less than 1.5 mm. Bruyere and colleagues [45] found tibial bone mineral density (BMD) as measured by dual x-ray absorptiometry to independently predict medial JSN over a one-year period in radiography, with patients in the lowest BMD quartile experience less JSN than those in the highest quartile. These observations are supported by our current MRI-based findings of increased cartilage loss in participants with subchondral sclerosis.

The greatest sensitivity to change (SRM) in this study was observed in subcohorts selected by baseline denuded area and initial cartilage thickness in MRI (up to -0.64). From the standpoint of recruiting patients for clinical trials, it must, however, be remembered that MRI is very costly and therefore cannot realistically be used as a screening tool. Using compartment-specific features of fixed flexion radiographs (specifically JSN and subchondral sclerosis), however, the SRM increased from -0.33 in the entire cohort to -0.47 for those with JSN grade 2 or 3 or participants with subchondral sclerosis. Such increases in SRM involve substantial savings in either the cohort size or the study duration in clinical trials and may thus justify the use of radiography as a screening tool in disease-modifying OA drug efficacy studies. Although it is currently unclear at which radiographic stage of radiographic OA disease-modifying drugs will be most effective, the current data can provide a reasonable basis for power calculations of the number or participants (with specific radiographic features) entered into a trial, if the effect of a disease-modifying OA drug is to be demonstrated.

## Conclusions

This study indicates that radiographic and MRI cartilage morphometry features of advanced disease (JSN, subchondral bone sclerosis, denuded bone areas, and low baseline cartilage thickness) appear to be associated with longitudinal cartilage loss in OA. Particularly radiography may be suited for selecting patients with a higher likelihood of fast progression in studies that try to demonstrate the cartilage preserving effect of disease-modifying OA drugs.

## Abbreviations

ANOVA: analysis of variance; BMD: bone mineral density; BMI: body mass index; ccMF: central aspect of the weight bearing medial femoral condyle; cMF: weight bearing medial femoral condyle; cMFTC: central medial femorotibial compartment; cMT: central medial tibia; FLASH: fast low angle shot; GLM: general linear models; JSN: joint space narrowing; JSW: joint space width; KLG: Kellgren Lawrence grade; MFTC: medial femorotibial compartment; mJSN: medial joint space narrowing; MRI: magnetic resonance imaging; MT: medial tibia; OA: osteoarthritis; SRM: standardized response mean; ThCtAB: change in cartilage thickness.

## Competing interests

FE is CEO of Chondrometrics GmbH, a company providing MRI analysis services. In the past five years, he has provided consulting services to Astra Zeneca, Chemedica, GlaxoSmithKline, MerckSerono, Nordo Nordisk, Pfizer, Virtualscopics, and Wyeth. SM, WW, and MH have part-time appointments with Chondrometrics GmbH. BW and M-PHLG are employed by Pfizer Inc The quantitative MRI analysis performed for this study was funded by Pfizer Inc.

## Authors' contributions

WW carried out the computation of the quantitative MRI endpoints. MH performed quality control of the MRI data and performed the conversion to a proprietary format. SM performed the quality control of all segmentations. WH and FE performed the statistical analysis. DH was one of the readers performing the radiographic readings. FE, BW, MN, MPH, and DH participated in the concept and design of the study. All authors were involved in writing the text and read and approved the final manuscript.
